# A Newly Discovered Constituent of the Blood

**Published:** 1898-04

**Authors:** 


### A Newly Discovered Constituent of the Blood.

Dr. Muller has given an account of certain objects, not hitherto described, which are to be found in every sample of blood, both in health and disease. They resemble fat globules, but must not be confounded with fragments of the white or red corpuscles; their size is variable, the largest diameter being 1-25,000 of an inch. They may be seen to show movement, and are not affected by osmic acid. Dr. Muller has named them "hemokonia" (blood dust).—*Ibid.*

